# Rare Diseases of the Anterior Segment of the Eye: Update on Diagnosis and Management

**DOI:** 10.1155/2015/947326

**Published:** 2015-09-27

**Authors:** Alessandro Lambiase, Flavio Mantelli, Marta Sacchetti, Siavash Rahimi, Giacomina Massaro-Giordano

**Affiliations:** ^1^Department of Sense Organs, Sapienza University of Rome, Viale del Policlinico, No. 155, 00196 Rome, Italy; ^2^Department of Biology, College of Science and Technology, Temple University, 1900 N. 12th Street, Philadelphia, PA 19122, USA; ^3^Cornea and Ocular Surface Unit, San Raffaele Hospital, Via Olgettina, No. 60, 20132 Milan, Italy; ^4^Queen Alexandra Hospital, Southwick Hill Road, Cosham PO6 3LY, UK; ^5^Scheie Eye Institute, University of Pennsylvania, 51 N. 39th Street, Philadelphia, PA 19104, USA

This special issue is focused on the current approaches used to identify and manage rare diseases of the anterior segment of the eye, which range from congenital to acquired disorders that are caused by ocular or systemic conditions and often have consequences that extend beyond the anterior segment of the eye. Specifically, the groups of Dr. R. Cascella et al. and Dr. G. L. Scuderi et al. provided a complete overview on primary congenital glaucoma and pediatric glaucoma, respectively, from diagnosis to treatment outcomes; the groups of Dr. D. Dobrowolski et al. and Dr. B. E. Ramírez et al. described the use of oral mucosa and stem cell therapy, respectively, for ocular surface reconstruction in rare diseases such as aniridia; Dr. F. Mantelli and colleagues reviewed the diagnosis and management of congenital corneal anesthesia and neurotrophic keratitis; Dr. A. K. Nowinska and colleagues compared different tomographic approaches to evaluate rare corneal dystrophies; the groups of Dr. M. Ołdak and Dr. S. P. Patel explained the pathophysiology of Fuchs endothelial corneal dystrophy and congenital hereditary endothelial corneal dystrophy, respectively; Dr. M. Sacchetti and colleagues provide a guide to diagnose and manage the iridocorneal endothelial (ICE) syndrome; Dr. S. Abdolrahimzadeh and colleagues described a series of rare diseases and ophthalmic alterations that may lead to glaucoma; Dr. M. F. Suárez et al. described how environmental agents can influence a rare corneal disease, climatic droplet keratopathy.

As can be seen from the variety of topics presented in this special issue, the disorders grouped under the umbrella term of rare diseases of the anterior segment of the eye can be considered quite heterogenous and numerous and, in turn, represent a substantial clinical burden on a worldwide basis [1]. In fact, although single disorders are rarely encountered in clinical practice, when taken together, they are not seldom seen. In addition, these diseases often represent a clinical challenge due to the paucity of specific diagnostic criteria and the lack of specific treatments. In fact, rare disorders tend to remain orphan drug indications due to the difficulty of running clinical trials, and surgical approaches are often inadequate due to concomitant anatomical and congenital anomalies.

To make the clinical picture of the rare diseases of the anterior segment of the eye even more difficult to manage, they often have consequences that extend beyond the anterior segment and can severely impair vision. Specifically, while corneal opacities and iris anomalies may directly affect visual function, several diseases such as iridocorneal angle anomalies may induce glaucoma with consequent damage to the optic nerve. In addition, several congenital diseases of the anterior segment of the eye are also associated with facial anomalies and other malformations of intraocular structures, making this group of disorders particularly complex for the ophthalmologist, who encounters obstacles in patients' management from diagnosis to treatment [2].

The recent advances in translational research, which are reshaping modern ophthalmology, allowed identifying the link between different clinical phenotypes and specific mutations in genes regulating the normal formation and maturation of the anterior segments of the eye [3]. In turn, our ability to understand the pathogenesis of rare and complex ophthalmic diseases has improved and, recently, novel treatments are being proposed for selected disorders of the anterior segment. For instance, biotechnology approaches for the pharmaceutical development of recombinant human molecules as well as novel procedures for ex vivo expansion of limbal stem cells may soon open novel therapeutic perspectives for patients with previously untreatable corneal disorders [4, 5]. Similarly, pre- and posttest genetic counseling plays an essential role in the achievement of an appropriate management of congenital glaucoma, in terms of medical, social, and psychological impact of the disease [6].

Nevertheless, due to the complexity of most of the rare anterior segment disorders of the eye, ranging from corneal disorders such as corneal dystrophies, neurotrophic keratitis, iridocorneal anomalies, and congenital glaucoma, further progress is mandatory to exert a significant impact on the natural history of the diseases [7–10]. Hopefully, the constant growth of novel diagnostic tools, accompanied by a better understanding of the disease pathophysiology, will also foster more preclinical and clinical research in this area of high medical need.

*Alessandro Lambiase* *Flavio Mantelli* *Marta Sacchetti* *Siavash Rahimi* *Giacomina Massaro-Giordano*
